# Spatial and Temporal High Processing of Visual and Auditory Stimuli in Cervical Dystonia

**DOI:** 10.3389/fneur.2017.00066

**Published:** 2017-03-03

**Authors:** Gaetana Chillemi, Alessandro Calamuneri, Francesca Morgante, Carmen Terranova, Vincenzo Rizzo, Paolo Girlanda, Maria Felice Ghilardi, Angelo Quartarone

**Affiliations:** ^1^Department of Clinical and Experimental Medicine, University of Messina, Messina, Italy; ^2^Department of Physiology, Pharmacology and Neuroscience, City University of New York Medical School, New York, NY, USA; ^3^Istituto Di Ricovero e Cura a Carattere Scientifico (IRCCS), Centro “Bonino Pulejo”, Messina, Italy; ^4^Department of Biomedical Science and Morphological and Functional Images, University of Messina, Messina, Italy

**Keywords:** Laterocollis, Torticollis, spatial processing, temporal processing, attention

## Abstract

**Objective:**

Investigation of spatial and temporal cognitive processing in idiopathic cervical dystonia (CD) by means of specific tasks based on perception in time and space domains of visual and auditory stimuli.

**Background:**

Previous psychophysiological studies have investigated temporal and spatial characteristics of neural processing of sensory stimuli (mainly somatosensorial and visual), whereas the definition of such processing at higher cognitive level has not been sufficiently addressed. The impairment of time and space processing is likely driven by basal ganglia dysfunction. However, other cortical and subcortical areas, including cerebellum, may also be involved.

**Methods:**

We tested 21 subjects with CD and 22 age-matched healthy controls with 4 recognition tasks exploring visuo-spatial, audio-spatial, visuo-temporal, and audio-temporal processing. Dystonic subjects were subdivided in three groups according to the head movement pattern type (lateral: Laterocollis, rotation: Torticollis) as well as the presence of tremor (Tremor).

**Results:**

We found significant alteration of spatial processing in Laterocollis subgroup compared to controls, whereas impairment of temporal processing was observed in Torticollis subgroup compared to controls.

**Conclusion:**

Our results suggest that dystonia is associated with a dysfunction of temporal and spatial processing for visual and auditory stimuli that could underlie the well-known abnormalities in sequence learning. Moreover, we suggest that different movement pattern type might lead to different dysfunctions at cognitive level within dystonic population.

## Introduction

Dystonia is a movement disorder characterized by patterned involuntary muscle contractions resulting in torsional movements and abnormal postures (1). Despite the “motor” definition of dystonia, there is increasing evidence that non-motor features, like depression (2) and dysfunctions in the sensory domain, are also present (3, 4). In keeping with this concept, few reports have shown subclinical sensory and perceptual dysfunctions (5) and impaired kinesthesia (6, 7); moreover, abnormal somatotopy in sensory areas has been reported by EEG (8), MEG (9–11), and fMRI (12, 13) studies. All these abnormalities are due to a dysfunction in sensory processing with a loss of lateral inhibition either in space or in time (13, 14). In fact, a series of studies have found that mild abnormalities in the primary sensory system are present in patients with dystonia both in spatial (15, 16) and temporal (17, 18) domains.

In dystonia, previous psychophysiological studies have related temporal and spatial dysfunctions as a consequence of sensory processing impairment, with a loss of lateral inhibition either in space or in time (13, 17, 19). However, the definition of such processing is controlled at higher cognitive level has not been sufficiently addressed. In addition, these studies have mostly focused on somatosensory stimuli finding out that somatosensory maps are alterated and produce blurred/altered representations of a person’s body in focal dystonia (20–22) with only a few studies investigating processing of visual stimuli (6, 23, 24), and no studies exploring processing of auditory stimuli.

There is increasing evidence that idiopathic cervical dystonia (CD) can be viewed as a circuit disorder, involving the basal ganglia-thalamo-cortical as well as cerebello-thalamo-cortical pathways (25, 26). The incorrect motor drive from the brain may cause various patterns of CD, the most common of which are the Laterocollis and the Torticollis; more rarely, we have dystonic tremor.

The aim of this study was to test whether in patients with CD the processing of spatial and temporal perception is impaired at higher cognitive level using recognition tasks that require attention to span both in the visual and auditory domains. Moreover, we further hypothesized that different movement pattern type of CD (Laterocollis, Torticollis, and prominent tremulous dystonia) may induce different cognitive deficit within dystonic population.

## Materials and Methods

Twenty-one subjects (W/M, 14/7) with idiopathic CD (mean age 55.2 ± 11.01 years) and 22 healthy controls (W/M, 11/10) (mean age 54.41 ± 12.1 years) were recruited at the Movement Disorders Centre of University of Messina. All subjects were right-handed according to Edinburgh Handedness Inventory. Dystonic population was subdivided in three subgroups according to the head movement pattern type derived from the prevalent subscore at the Tsui scale (27): eight subjects were diagnosed as Laterocollis, eight were diagnosed as Torticollis, and five were diagnosed as tremulous CD (Tremor). All patients underwent extensive neurological examination and laboratory and neuroimaging investigations to rule out acquired causes of dystonia. None of the enrolled patients has been ever treated with drugs blocking the dopamine receptor. All drugs affecting the central nervous system were discontinued at least 1 week prior to the study; all patients were receiving botulinum toxin injections and were examined at least 3 months after the last injection. Clinical features of dystonic patients are given in Table 1.

**Table 1:** **Clinical details of cervical dystonia subjects recruited for this study**.

Subject	Age	Gender	Disease duration (years)	Toxin type injection	Toxin therapy duration (years)	Pattern (primary component)
S1	56	F	3	OnabotulinumtoxinA	1	Laterocollis
S2	61	F	13	OnabotulinumtoxinA	13	Torticollis
S3	45	F	22	OnabotulinumtoxinA	6	Tremor
S4	26	M	9	AbobotulinumtoxinA	8	Laterocollis
S5	58	M	36	AbobotulinumtoxinA	22	Torticollis
S6	66	F	16	OnabotulinumtoxinA	3	Laterocollis
S7	38	F	18	OnabotulinumtoxinA	3	Tremor
S8	46	M	33	AbobotulinumtoxinA	20	Laterocollis
S9	71	F	7	AbobotulinumtoxinA	6	Torticollis
S10	52	F	14	AbobotulinumtoxinA	7	Torticollis
S11	53	F	14	AbobotulinumtoxinA	9	Laterocollis
S12	62	F	13	OnabotulinumtoxinA	6	Laterocollis
S13	66	F	18	AbobotulinumtoxinA	18	Torticollis
S14	64	M	4	AbobotulinumtoxinA	3	Torticollis
S15	61	F	14	OnabotulinumtoxinA	12	Torticollis
S16	64	F	12	AbobotulinumtoxinA	12	Laterocollis
S17	50	M	27	AbobotulinumtoxinA	27	Torticollis
S18	46	M	9	AbobotulinumtoxinA	6	Laterocollis
S19	67	M	2	AbobotulinumtoxinA	1	Laterocollis
S20	61	F	16	OnabotulinumtoxinA	13	Tremor
S21	47	F	36	OnabotulinumtoxinA	16	Torticollis

### Ethical Approval

The local ethical committee approved the entire research protocol and all subjects signed an informed consent before examination.

### Task Settings

**Figure 1:**
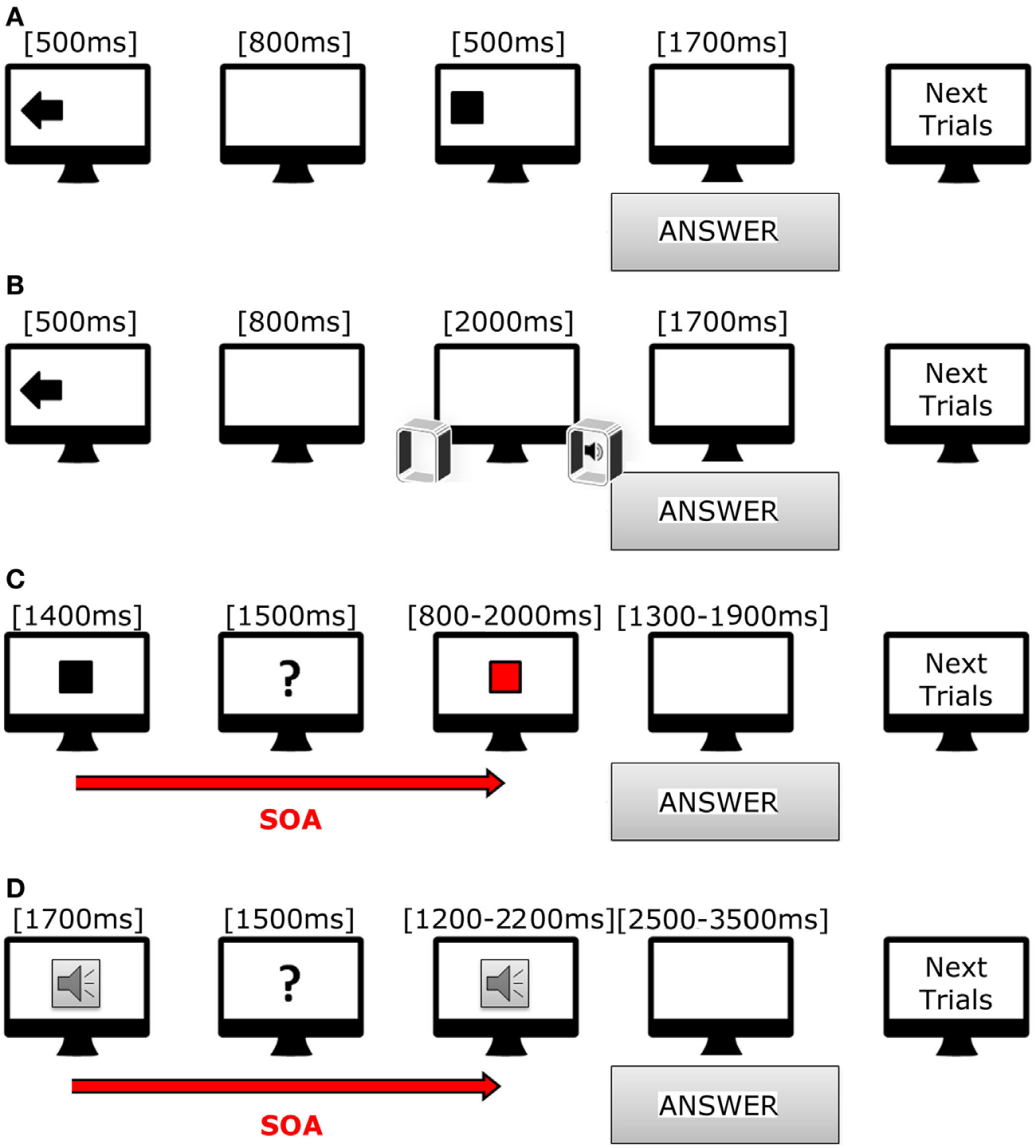
**Cartoon showing trials used in our tasks**. **(A)** Scheme of incongruent trial in visual–spatial task. **(B)** Scheme of congruent trial in acoustic-spatial task. **(C)** Scheme of short trial in visual–temporal task. **(D)** Scheme of long trial in acoustic-temporal task.

All tasks were prepared with Psychopy software (28) release 1.81. Stimuli were presented with a Dell workstation (21″ Dell monitor, resolution 1,680 × 1,050 pixels), and two speakers that were equidistant from the monitor. Subjects were comfortably sitting in front of the monitor at a distance of 70 cm (Figure 1).

### Spatial Recognition Tasks

Each subject was asked to identify the position of either visual or auditory stimuli at a constant intertrial interval of 3,500 ms.

In the *visuo-spatial* task (Figure 1A), target presentation was preceded by the appearance of a visual cue lasting for 500 ms; the visual cue consisted of an arrow pointing either in right/left or down/up direction, or a cross in the center of the screen. After 800 ms, a white square (190 × 190 pixels) appeared for 500 ms in one of the four quadrants of the screen (right or left, upper and inferior). Subjects had to indicate as fast as possible the target position with respect to the direction of the preceding using a keyboard. The responses were thus classified in six categories: congruent (same direction for target and arrow), incongruent (opposite direction for target and arrow), neutral (target preceded by a cross). A total of 145 trials were presented (8.46 min).

In the *audio-spatial* task (Figure 1B), the target sound was preceded by the appearance of a visual cue on the screen, namely, an arrow pointing either to the right or left as well as a cross on screen center that lasted for 500 ms. The target sound was a beep (WAV, 44 kHz, 16 bit, duration 2,000 ms) produced by either the right or left speaker or simultaneously by both speakers. Subjects had to answer as soon as they realized the sound position using the same keyboard. The responses were thus classified in six categories: congruent (same direction for sound and arrow), incongruent (opposite directions for sound and arrow), neutral (sound preceded by a cross). We presented 72 trials (duration 4.2 min).

### Temporal Recognition Tasks

The subjects had to evaluate whether the duration of a target stimulus, visual or auditory, was different from that of a reference stimulus. In the *visuo-temporal* task (Figure 1C), the reference stimulus was a white square appearing at the center of the screen for either 1,400 ms (fast condition) or 1,800 ms (slow condition). After 1,500 ms the target stimulus was presented, namely a red square appearing in the same position as the reference one. The target stimulus was either fast (range from 800 to 2,000 ms, with the reference stimulus of 1,400 ms) or slow (range from 1,000 to 2,600 ms, with the reference stimulus of 1,800 ms). At the end of each trial, each subject had to decide whether the duration of the target stimulus was equal to, shorter or longer than the reference. They were asked to answer as soon as possible after target presentation by using a keyboard. The responses were again classified in six categories: fast-shorter, fast-equal, fast-longer, slow-shorter, slow-equal, and slow-longer. A total of 72 trials were presented (8.34 min).

In the *audio-temporal task* (Figure 1D), the reference stimulus, a beep tone (WAV, 44 kHz, 16 bit), was presented for either 700 ms (fast) or 1,700 ms (slow), arranged in line with a screen one to the right and the other to the left. After 1,500 ms, the target stimulus was presented. During the *fast* condition, the target stimuli duration ranged from 400 to 1,000 ms (reference duration 700 ms), while during the *slow* condition, duration ranged from 1,200 to 2,200 ms (reference duration 1,700 ms). A total of 72 trials divided in 2 section were presented (8.12 min).

### Data Analysis

For all the four tasks, we measured accuracy (number of correct answers) and reaction times (interval between stimulus onset and response).

Linear mixed models (LMM) were run separately on spatial and temporal tasks using accuracy rates as dependent variable to investigate differences between controls and different forms of dystonia (PatternType). For LMM on spatial tasks, three within-subject factors were considered: type of Stimulus (visual vs auditory), Congruency (congruent vs incongruent vs uninformative), and Position (right vs left vs centered). Notice that, for visual task, performances related to stimuli presented either in the upmost or in the bottom part of the screen were averaged together to facilitate comparison with the “centered” condition of auditory task; this decision was taken after observing that results from those two conditions largely overlapped both in terms of accuracy and reaction times.

For LMM on temporal tasks, following within-subject factors were considered: type of Stimulus (visual vs auditory), Speed (fast vs slow), Duration (shorter-shorter vs shorter vs equal vs longer vs longer-longer).

In both models, age and gender were used as covariates. As our primary interest was investigating accuracy rates, we further included an interaction between PatternType and reaction time as a covariate in the models; in this way, we wanted to account for potential interaction effects, e.g., speed-accuracy trade-off. In LMMs, covariance structure of residuals related to repeated measurement is explicitly fitted together with parameter estimates; different structures were adopted, and compared by means of Akaike information criteria (AIC). The smaller the AIC the better the model fit (29, 30). For both spatial and temporal LMMs, the best performances were obtained using a heterogeneous Toeplitz structure. Random effect on subjects was not included in the final models as the percentage of variance explained in this way was negligible after modeling covariance structure of residuals. In all analyses, cutoff for significance was set to 0.05. Multiple comparisons issue was accounted for by applying Bonferroni correction.

Before being administered cognitive tasks, Tsui and Pain scales were determined on CDs. At the time of the study, they were receiving different toxin types; moreover, disease duration and toxin therapy duration were quite variable. Thus, we tested whether those variables may influence either accuracy rates or reaction times. To this end, for both accuracy rates and reaction times, we estimated Kendall’s tau correlation coefficients between those measures and disease duration, as well as toxin therapy duration, Tsui and Pain scales. This analysis was performed both on global measures obtained by pooling all tasks together, as well as for each separate task. Moreover, we tested whether systematic differences may exist due to toxin type injection.

## Results

Table 1 shows the clinical features of dystonic patients. Correlation analyses performed between task outcomes and clinical features did not show significant differences within dystonic group, neither when pooling al subjects together, nor when separately analyzing subjects belonging to different dystonic patterns (corrected *p*-values >0.05).

### Spatial Tasks

**Figure 2:**
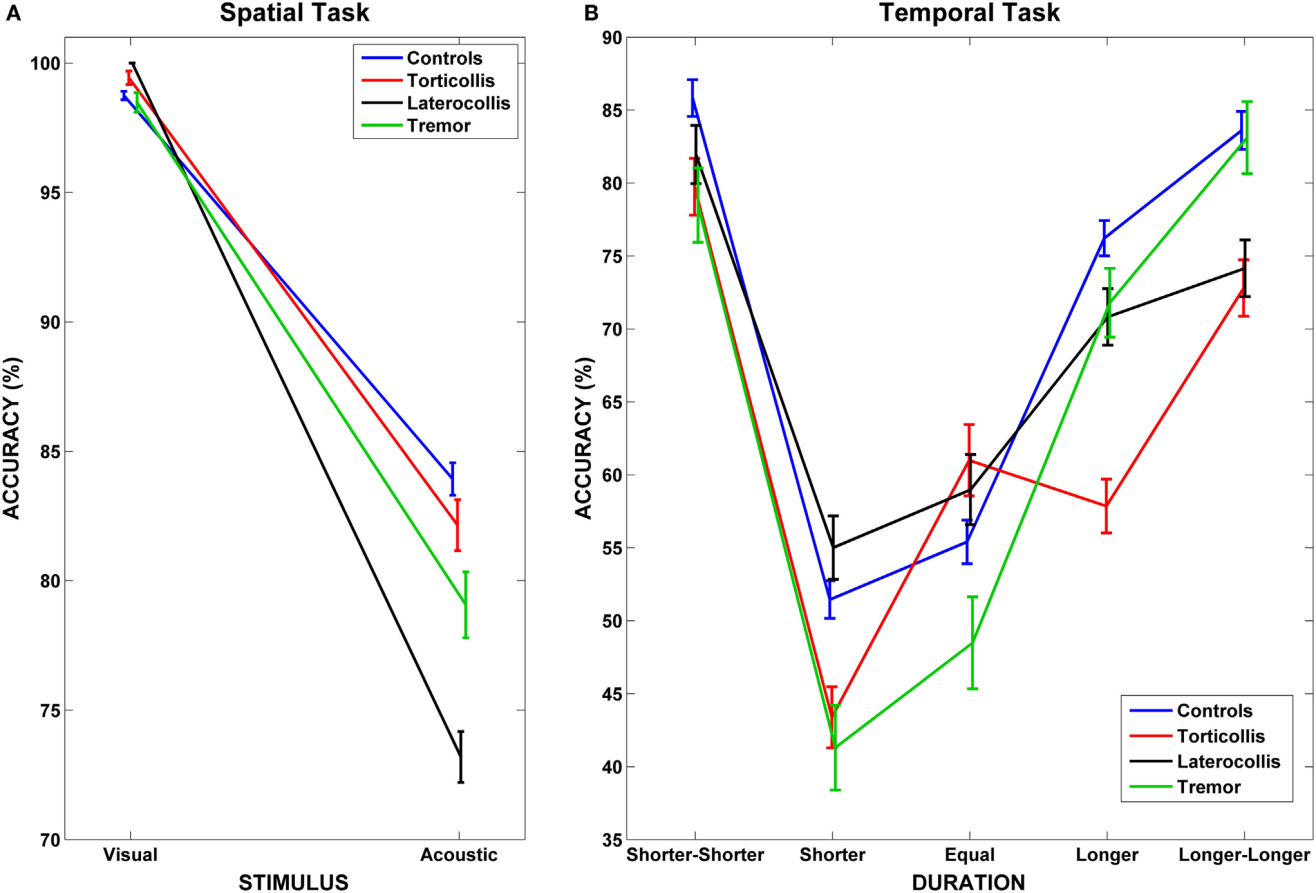
**Interaction between dystonic patients and tasks**. **(A)** Accuracy rates for controls and dystonic subtypes highlight the lower accuracy for Laterocollis with respect to controls when spatially detecting acoustic stimuli. **(B)** We observe decreased accuracy for Torticollis with respect to controls when detecting target sounds that lasted “longer” than reference sounds in temporal tasks. Bars represent SEM.

Results of LMM performed on spatial tasks are reported in detail in Table 2. A main effect of PatternType was observed (*F* = 22.404, *p* < 0.001); however, *post hoc* comparisons did not reveal significant differences after correcting for multiple comparisons. Furthermore, a significant interaction between PatternType and Stimulus was observed (*F* = 5.431, *p* = 0.002); *post hoc* analyses revealed that subjects diagnosed as Laterocollis were significantly less accurate than controls when processing auditory stimuli (average diff = 10.75%, SD error = 3.502, corrected *p* = 0.021, see Figure 2A). Moreover, a significant interaction with reaction time was observed (*F* = 36.989, *p* < 0.001). *Post hoc* analyses, after applying Bonferroni correction, showed that subjects diagnosed as Torticollis and Laterocollis were consistently slower than controls (*Z* = −2.976, corrected *p* = 0.018 and *Z* = −3.702, corrected *p* = 0.001, respectively). No significant differences were detected between controls and Tremor group, likely due to lack of statistical power.

**Table 2:** **Results of linear mixed models applied on spatial tasks**.

Factors included in the model	*F*	*p*-Value
PatternType	22.404	<0.001
Stimulus	224.248	<0.001
Congruency	38.834	<0.001
Position	121.565	<0.001
PatternType*Stimulus	5.431	0.002
PatternType*Congruency	1.531	0.171
PatternType*Position	0.733	0.624
Stimulus*Congruency	38.449	<0.001
Stimulus*Position	122.449	<0.001
Congruency*Position	59.004	<0.001
PatternType*RT	36.989	<0.001

*For each factor included in the model, estimated *F* statistics and obtained *p*-value were reported. For interactions included in the model, factors used were reported and linked *via* an asterisk. Significative components were underlined. RT = reaction time; PatternType = variable coding healthy group and dystonic subjects subgroups; Stimulus = variable coding stimuli used in the task (visual and auditory); Congruency = variable coding congruency levels (congruent vs incongruent vs uninformative); Position = variable coding target positions (right vs left vs centered)*.

### Temporal Tasks

Results of LMM performed on temporal tasks are reported in detail in Table 3. We found a significant interaction between PatternType and Duration factors (*F* = 2.585, *p* = 0.003); *post hoc* analyses revealed, after applying Bonferroni correction, that subjects diagnosed as Torticollis were significantly less accurate than controls when detecting target stimuli whose duration with respect to reference sound was in condition *longer* (average diff = 18.362%, SD error = 6.616, corrected *p* = 0.043, see Figure 2B). Unlike for the spatial task, no significant interaction was observed with reaction times.

**Table 3:** **Results of linear mixed models applied on temporal tasks**.

Factors included in the model	*F*	*p*-Value
PatternType	0.067	0.977
Stimulus	0.090	0.765
Speed	0.329	0.567
Duration	31.700	<0.001
PatternType*Stimulus	0.187	0.905
PatternType*Speed	1.078	0.358
PatternType*Duration	2.585	0.003
Stimulus*Speed	6.993	0.008
Stimulus*Duration	5.664	<0.001
Speed*Duration	6.775	<0.001
PatternType*RT	0.592	0.669

*For each factor included in the model, estimated *F* statistics and obtained *p*-value were reported. For interactions included in the model, factors used were reported and linked *via* an asterisk. Significative components were underlined. RT = reaction time; PatternType = variable coding healthy group and dystonic subjects subgroups; Stimulus = variable coding stimuli used in the task (visual and auditory); Congruency = variable coding congruency levels (congruent vs incongruent vs uninformative); Position = variable coding target positions (right vs left vs centered)*.

## Discussion

Dystonia is a movement disorder characterized by patterned voluntary muscle contractions (1). However, non-motor features are also present, which may arise as a consequence of motor impairment or might have a more inherent genetic basis (2–4, 31–33). In this study, we aimed to test whether, in patients with idiopathic CD, the processing of spatial and temporal perception is impaired at higher cognitive level. We found that CD patients showed perceptive dysfunctions of different level with respect to neurophysiologic pattern type: a double dissociation in the performance between Laterocollis and Torticollis undergoing spatial and temporal tasks was indeed detected.

### Spatial Tasks

In visuo-spatial task, we observed that dystonic patients were on average slower than controls, although significant differences were detected only for Torticollis and Laterocollis subtypes. This result likely reflects the motor features of the disorder (34). When investigating accuracies in spatial tasks, we found that Laterocollis were worse than healthy subjects in auditory but not visual task. It is known that a sound lasts in short-term memory for about 4 s while a visual stimulus persists for only 0.25 s (35); thus, we may hypothesize that the first sound mask the second one interfering on the response accuracy of the second auditory stimulus. This result may therefore indicate a perceptual abnormality on auditory recognition for Laterocollis.

Recent studies using intracranial recordings (36) and functional imaging (37) have provided compelling evidence for a hemispheric specialization in the auditory processing. It has been suggested that the processes associated with identification of linguistic auditory objects are lateralized toward the left hemisphere (38) while the paralinguistic aspects of vocal processing are lateralized toward the right hemisphere (39). According to this theory, in the left hemisphere, anterior brain structures are involved in expressive tasks, whereas posterior areas contribute to stimulus perception (40).

It is well known that idiopathic dystonia is mainly attributed to basal ganglia dysfunction (11). Hence, a greater left damage involving these structures may explain perceptual abnormality observed on the performance of the Laterocollis. It is important to highlight that this result was not confirmed neither for Torticollis, who were on average 1.78% less accurate than controls (SD error 3.5%), nor for subjects diagnosed as Tremor, who were on average 4.86% less accurate than controls (SD error 4.42%).

### Temporal Tasks

When investigating temporal tasks, we found that subjects diagnosed as Torticollis were less accurate than healthy subjects when the duration of stimulus (exposure times) was in the condition “*longer*” with respect to reference sound.

It is supposed that the representation of numbers is arranged along a mental line, called mental number line (MNL) (41, 42). In people who read from left to right, the MNL is spatially oriented from left to right (43); Moreover, it is known that two numbers that are numerically distant are more easily and quickly detected [distance effect (44)]. In accordance to these theories, we observed that for all subjects it was easier to temporally discriminate reference from target sounds in presence of a clear temporal difference (45). This situation can be visualized in Figure 2B when looking at “*shorter-shorter*” and “*longer-longer*” conditions. The condition “*equal*” was the most difficult to detect, while performances improved either when moving towards “*shorter*” or “*longer*” conditions. Of importance, such pattern did not hold for Torticollis, who did not show an advantage for “*long*” condition (see Results).

Shomstein and Behrmann (46) found that longer exposure times of stimuli allowed more time for perceptual grouping and figure-ground segmentation, leading to the best representation of the object. On other hand, the effects of this representation diminished with long SOA (time between the onsets of reference and target components). This effect may explain the loss of accuracy of Torticollis in our temporal task. Therefore, Torticollis’ increased likelihood of making mistakes as time interval increases might undergo a difficult in maintaining temporal object representation. Some evidence of abnormal timing adjustment in CD was observed elsewhere (47, 48). However, in Filip et al. (48), both Torticollis and Laterocollis were not explicitly compared against each other: it might be the case that it was the Torticollis subgroup to mostly drive their result.

### Pathophysiological Correlates of Visuo-Spatial and Audio-Spatial Impairment in CD

Several abnormalities in posterior parietal cortex (PPC) and ventral frontoparietal circuits have been observed in clinically unaffected carriers of the DYT1 dystonia mutation during learning of visuo-motor sequences, with a compensatory increased activation in the left ventral prefrontal cortex and lateral cerebellum (21). Furthermore, abnormal connectivity between PPC and primary motor cortex (M1) may be present in CD, an abnormality that is associated with slower reaching movements (49, 50). The interaction between PPC and M1 is crucial for the preparation and planning of movements directed to visual targets (51, 52), as it regulates visuo-spatial mechanisms that affect performance, accuracy, and variability.

The differences observed in our study between Laterocollis and Torticollis might be explained by involvement of different brain structures in these groups of patients, confirming a pivotal role of these circuits in the pathophysiology of idiopathic dystonia.

Indeed, a consistent body literature supports a major role of the basal ganglia in dystonia; however, more recent findings explore the causative role of other regions, in particular the cerebellum (48, 53), but no previous study has linked the respective basal ganglia and cerebellar networks with different patterns of dystonia. We might hypothesize that the difficulty of identifying the stimuli as result of spatial attention deficits, as we found in the case of Laterocollis, may be more marked in dystonic patients with major impairment of the basal ganglia. On the other side, it may be supposed that problems in time estimation of stimuli, as we found in Torticollis, are due to difficulty of object-based selective attention when a major cerebellar involvement occurs.

We did not identify, within CD group and after correcting for multiple comparisons, significant correlations between measured task outcomes and clinical features. It is, however, worth to mention that average accuracy rates in audio-spatial task showed a negative correlation with Tsui scale (Kendall’s tau = −0.408, uncorrected *p* = 0.016); this result did not come unexpected and may reflect increased difficulty for CDs to achieve good accuracy in audio-spatial processing when degree of impairment increases.

Finally, it may be postulated that the abnormal auditory and visual processing might also have a reflection on movement programming and planning (54). Movement preparation is known to be impaired in dystonia; movement preparation involves a number of factors, including the process of sensorimotor integration (55). The human nervous system prefers to be anticipatory rather than reactive, and a disordered preparation for movement will certainly be a crucial factor in faulty execution.

The heterogeneity and the small size of the population under study have to be considered the limitations of the present work. In addition, due to the small number of subjects, we could not stratify the patients according to the side (i.e., right or left) of the affected muscles. Future studies will be necessary to better explore parieto-motor connectivity during audio-motor or visuo-motor tasks by means, for instance, of fMRI or dual-coil TMS approaches, and to further clarify the differences, we observed within subtypes of dystonia.

## Author Contributions

GC: substantial contributions to the conception and design of the work, drafting the work; agreement to be accountable for all aspects of the work in ensuring that questions related to the accuracy or integrity of any part of the work are appropriately investigated and resolved. AC: substantial contributions to the conception and design of the work; responsible for data analyses; and substantial contribution to creation of figures for the work. FM: substantial contributions to the conception and design of the work, acquisition and interpretation of data for the work; agreement to be accountable for all aspects of the work in ensuring that questions related to the accuracy or integrity of any part of the work are appropriately investigated and resolved. CT: substantial contributions to the interpretation of data for the work; revising the work critically for important intellectual content; and agreement to be accountable for all aspects of the work in ensuring that questions related to the accuracy or integrity of any part of the work are appropriately investigated and resolved. VR: substantial contributions to the interpretation of data for the work, drafting and revising the work critically for important intellectual content. PG: substantial contributions to the conception or design of the work; agreement to be accountable for all aspects of the work in ensuring that questions related to the accuracy or integrity of any part of the work are appropriately investigated and resolved; and revising the work critically for important intellectual content. MG: substantial contributions to revising the work critically for important intellectual content; final approval of the version to be published. AQ: substantial contributions to the conception and design of the work, drafting the work; agreement to be accountable for all aspects of the work in ensuring that questions related to the accuracy or integrity of any part of the work are appropriately investigated and resolved; and final approval of the version to be published.

## Conflict of Interest Statement

The authors declare that the research was conducted in the absence of any commercial or financial relationships that could be construed as a potential conflict of interest.
